# Co-delivery of sorafenib and crizotinib encapsulated with polymeric nanoparticles for the treatment of *in vivo* lung cancer animal model

**DOI:** 10.1080/10717544.2021.1979129

**Published:** 2021-10-05

**Authors:** Tian Zhong, Xingren Liu, Hongmin Li, Jing Zhang

**Affiliations:** a Department of Pulmonary and Critical Care Medicine, Sichuan Provincial People's Hospital, University of Electronic Science and Technology of China (Chinese Academy of Sciences Sichuan Translational Medicine Research Hospital), Chengdu, China; b Tumor Center, Sichuan Provincial People's Hospital, University of Electronic Science and Technology of China (Chinese Academy of Sciences Sichuan Translational Medicine Research Hospital), Chengdu, China

**Keywords:** Polymeric nanoparticles, co-delivery, lung cancer, apoptosis, *in vivo* animal model

## Abstract

To treat various cancers, including lung cancer, chemotherapy requires the systematic administering of chemotherapy. The chemotherapeutic effectiveness of anticancer drugs has been enhanced by polymer nanoparticles (NPs), according to new findings. As an outcome, we have developed biodegradable triblock poly(ethylene glycol)–poly(ε-caprolactone)–poly(ethylene glycol) (PEG–PCL–PEG, PECE) polymeric NPs for the co-delivery of sorafenib (SORA) and crizotinib (CRIZ) and investigated their effect on lung cancer by *in vitro* and *in vivo*. There is little polydispersity in the SORA–CRIZ@NPs, an average size of 30.45 ± 2.89 nm range. A steady release of SORA and CRIZ was observed, with no burst impact. The apoptosis rate of SORA–CRIZ@NPs was greater than that of free drugs in 4T1 and A549 cells. Further, *in vitro* cytotoxicity of the polymeric NPs loaded with potential anticancer drugs was more quickly absorbed by cancer cells. On the other hand, compared to free drugs (SORA + CRIZ), SORA + CRIZ@NPs showed a substantial reduction of tumor development, longer survival rate, and a lowered side effect when delivered intravenously to nude mice xenograft model with 4T1 cancer cells. TUNEL positivity was also increased in tumor cells treated with SORA–CRIZ@NPs, demonstrating the therapeutic effectiveness. SORA–CRIZ@NPs might be used to treat lung cancer soon, based on the results from our new findings.

## 1. Introduction

Due to cancer-related mortality, 25% are attributable to lung cancer; this proportion is higher than the combined sum of lung, colon, and prostate cancers together. Fast 50% of lung cancer cases are diagnosed in stage IV, with a dismal probability of survival. This is due to a lack of early screening testing (Liu et al., 2014; Chen et al., 2015; Novoselova et al., 2020). Further complicating the treatment process is that traditional treatments cannot reach the deeper parts of the lung. Small cell lung cancer (SCLC) and non-small cell lung cancer (NSCLC) are the two primary forms of lung cancer (NSCLC). Left untreated, the median survival time for SCLC is four months (Yuan et al., 2018; Abdelaziz et al., 2020; de Menezes et al., 2021). The quick growth rate, early metastasis, and fast metabolism are the leading causes of its high lethality. A neuroendocrine tumor, SCLC, is filled with neurosecretory vesicles and neurofilaments, which are the hallmarks of this cancer type (Keller et al., 2011). Nearly, 80–85% of lung cancers are caused by this kind of cancer, which is not amenable to standard chemotherapy and radiation (Kaplan et al., 2016). Cancers of the epidermis, big cells, broncho-alveolar organs, and adenocarcinomas can also be categorized as NSCLC. Every single one of these NSCLC subtypes is unique and reacts to specific treatments differently (Chen et al., 2021).

A number of these technologies can be applied to gliomas, and nanobiotechnology substantially contributes to drug delivery in cancer. Scientists are working to develop nanoparticles (NPs) that can cross the blood–brain/blood–tumor barrier, be taken up by glioma cells specifically and differentially, and release their payload on an extensive period to achieve a clinical response (Liu et al., 2017; Xu et al., 2019; Sun et al., 2021). Due to their safety in the clinic, NPs derived from poly(d,l-lactic-co-glycolic acid) (PLGA) are an excellent choice. If nanocarriers are passively targeted to the tumor by their enhanced permeability and retention (EPR), PEG-functionalized PLGA NPs are particularly desirable (Başpınar et al., 2019; Li et al., 2020; Norouzi & Hardy, 2021). Pegylated polymeric NPs have drastically decreased systemic clearance compared to similar NPs without PEG. Due to the development of biotechnology, chemotherapeutic agents can be delivered directly to the tumor site using targeting ligands that bind to biological molecules or receptors on the glioma cells (Sun et al., 2020; Zang et al., 2021; Zhang et al., 2021).

**Figure 1.**
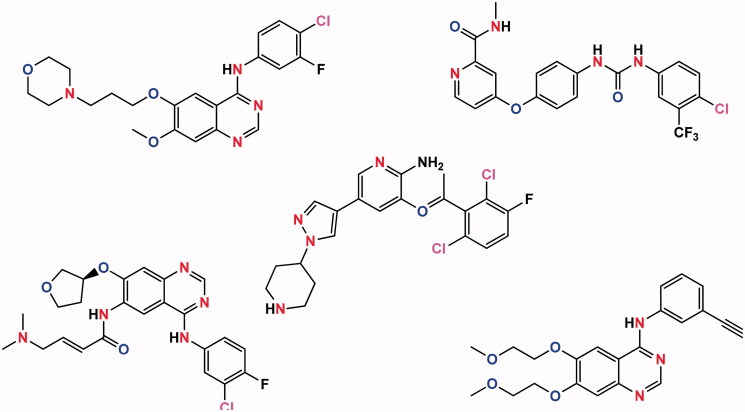
Molecular structure of some of the potential anticancer compounds.

Perfusion coefficients for FDA approved anticancer drugs (afatinib, sunitinib, sorafenib (SORA), gefitinib, crizotinib (CRIZ), and erlotinib) (Figure 1) were determined using a system called the Transwell (Chang et al., 2015). Inhibitors of the epidermal growth factor receptor (EGFR) tyrosine kinase, gefitinib, and erlotinib, are first-generation small molecule inhibitors. An inhibitor of EGFR, afatinib is a second-generation small-molecule inhibitor that also targets the EGFR pathway NSCLC adenocarcinomas with activating EGFR mutations that are eligible for therapy with these drugs. Orally active multikinase inhibitors, SORA and sunitinib, have been authorized to treat hepatocellular carcinoma and renal cell carcinoma (Loira-Pastoriza et al., 2021; Viswanadh et al., 2021; Xiao et al., 2021). ALK/cMET inhibitor CRIZ has been approved for use in NSCLC, while dasatinib is approved for chronic myeloid leukemia in a selected population. Intestinal uptake of these compounds is determined by the physicochemical properties of these compounds or by their ability to categorize in cellular organelles and cause resistance (Malik & Mukherjee, 2018).

**Figure 2.**
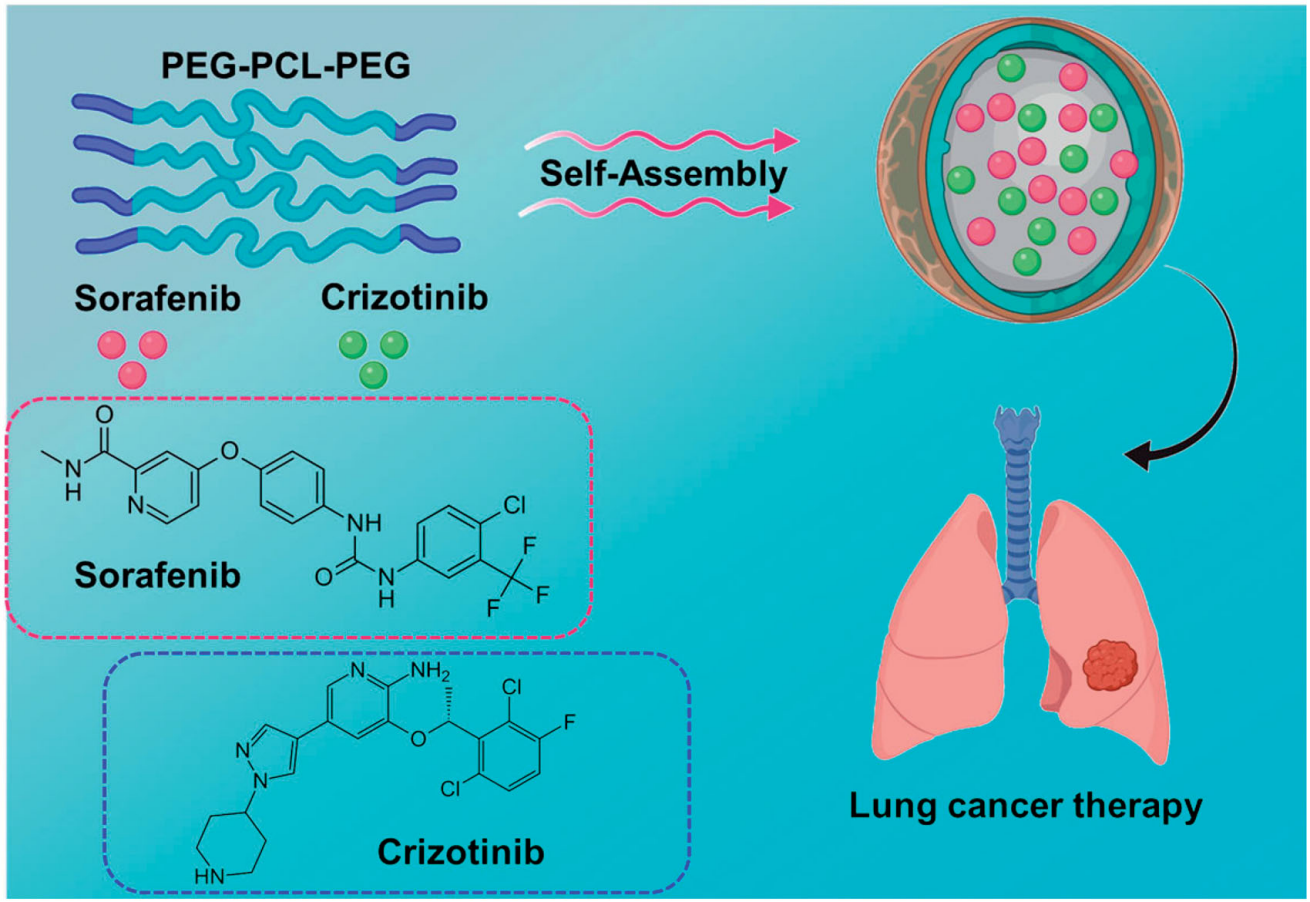
Construction of SORA–CRIZ@NPs. Triblock poly(ethylene glycol)–poly(ε-caprolactone)–poly(ethylene glycol) (PEG–PCL–PEG, PECE) polymeric nanoparticles (NPs) for the co-delivery of sorafenib (SORA) and crizotinib (CRIZ) and investigated their effect on lung cancer by *in vitro* and *in vivo*.

In this present investigation, we have effectively fabricated the biodegradable triblock poly(ethylene glycol)–poly(ε-caprolactone)–poly(ethylene glycol) (PEG–PCL–PEG, PECE) and utilized to establish nanoparticulate for drug delivery system of SORA–CRIZ@NPs for the active dual-delivery of SORA and CRIZ (Figure 2). This polymer comprises a hydrophilic PEG block and a hydrophobic PCL block, and its form into the linear polyester polymer. SORA and CRIZ show that water solubility may be increased by using PCEC as a promising nanocarrier, and their therapeutic efficacy against lung tumors might have improved. In addition, these nanoparticulate shows less toxic, cytocompatibility, good biocompatibility, and amphiphilic features, PECE polymeric NPs were selected to develop a drug delivery system.

## 2. Experimental

### 2.1. Preparation of SORA–CRIZ@NPs

Sorafenib and CRIZ (≥99%) were obtained from J&K Chemical Technology (Beijing, China). ε-caprolactone (ε-CL), poly(ethylene glycol) (PEG, Mn = 2000), and [Sn(Oct)2] were purchased from GL Biochem (Shanghai, China). All commercially available chemicals were used without further purification. Roswell Park Memorial Institute Medium (RPMI-1640), fetal bovine serum (FBS), penicillin/streptomycin, 3-(4,5-dimethylthiazol-2-yl)-2,5-diphenyl tetrazolium bromide (MTT), AO/EB, and DAPI staining solution were obtained from Gibco Corporation (Carlsbad, CA).

4T1, A549, and non-cancerous HUVEC cells (a human lung cancer cells, American Type Culture Collection, Manassas, VA) were obtained from the Cell Bank of Chinese Academy of Sciences (Shanghai, China). 4T1 and A549 cancer cells were cultured in Roswell Park Memorial Institute Medium (RPMI-1640) (Gibco, Carlsbad, CA) with 10% FBS and 1% of streptomycin and penicillin addition under a 5% CO_2_ atmosphere at 37 °C.

Nude mice-bearing 4T1 xenograft model was established as animal models for *in vivo* examination. The mice were supplied by the Beijing Vital River Laboratory Animal Technology Co., Ltd. (Beijing, China) and maintained under specific pathogen-free conditions and cared for and treated under standard protocols approved and supervised by the Chinese Academy of Sciences Sichuan Translational Medicine Research Hospital Experimental Ethics Committee.

### 2.2. Fabrication of SORA–CRIZ@NPs

In a previous report (Xu et al., 2015; Xiong et al., 2020; Jangid et al., 2021), PECE polymer (MW = 3700) was fabricated via the ring-opening polymerization reaction method. To prepare the SORA–CRIZ@NPs, 40 mg SORA and 40 mg CRIZ were assessed and combined in 10 mL of methanolic solution before being added to the same reaction mixture. SORA–CRIZ were added to a 15 mL centrifuge tube at various ratios (2:1, 1:1, 1:2; SORA 2 mg, 3 mg, 4 mg; CRIZ 4 mg, 3 mg). PECE polymer was weighed and mixed in dichloromethane to yield 28 mg, dissolved in 4 mL. The reddish-orange color was obtained after the hybrid systems were combined uniformly. These two hybrid systems were then combined in a rotary evaporator, and the organic solvent was removed. Water heated to 60 °C was used to wash the film. A 0.22 µm filter was used to remove non-encapsulated SORA and CRIZ from the fabricated reaction solution. SORA–CRIZ@NPs powder was obtained by freezing the filtrate.

### 2.3. Characterization of polymeric nanoparticles

Transmission electron microscopic (TEM) images of SORA–CRIZ@NPs were taken with a JEM-2010HR transmission electron microscope (JEOL, Tokyo, Japan). The hydrodynamic size (*z*-average), polydispersity index (PDI), and zeta-potential (*ζ*-potential) distributions of the SORA–CRIZ@NPs were measured by Zetasizer Nano ZS (Malvern Instruments Ltd., Worcestershire, UK). The relevant data were recorded as the average of three measurements.

### 2.4. Drug loading (DLs), entrapment efficiencies (EEs), and *in vitro* drug release study

We measured the DLs and EEs of SORA–CRIZ@NPs by HPLC (Zorbax C18, Agilent, Santa Clara, CA). The column used for the detection was C_18_ (4.6 × 100 mm × 3.5 mm, and the mobile phase used consisted of 0.2 M of Na_2_HPO_4_:acetonitrile (90:10, v/v) and 0.2 M citric acid (2:1) with the flow rate of 1.0 mL/min. The temperature of the wavelength and column used for drug detection were 302 nm and 25 °C, respectively. In the previous report, DLR and EE of SORA–CRIZ@NPs were calculated based on those values.

*In vitro* drug release of SORA and CRIZ from SORA–CRIZ@NPs used the dynamic dialysis techniques. A solution of SORA and CRIZ was prepared by dissolving both compounds in DMSO. Deionized water was used to dissolve the SORA–CRIZ@NPs and then put into dialysis bags. Tween-80 (0.5%, w/w) was added to the centrifuge tube holding the dialysis bags (100 rpm). Centrifuged at 13,000 rpm for 30 min at 4 °C, the collected medium was centrifuged for at least 30 min. It was then replaced with 2 mL of the newly warmed medium at the same time each day. HPLC has been used to examine the supernatant, as reported earlier (Cho et al., 2006; Nurunnabi et al., 2015; Sun et al., 2018).

### 2.5. *In vitro* cellular uptake

1 × 10^6^ cells per well were plated in six-well plates for 24 h. Saline, a mixture of SORA and CRIZ powders and SORA–CRIZ@NPs, was added to the 4T1 and A549 cells for 2 h. We next examined the fluorescence signals using a fluorescence microscope and washed the cells using PBS (pH 7.4, 0.01 M) before examining the fluorescence (Leica inverted Fluorescence Microscope SP5 II HCS A, Wetzlar, Germany). The fluorescence features were used to study the cellular uptake in 4T1 and A549 cells.

### 2.6. *In vitro* cytotoxicity assay

4T1 and A549 cells were treated with blank PECE, free drugs (SORA + CRIZ) and the SORA–CRIZ@NPs using MTT assay to assess cytotoxicity. It was dissolved in DMSO, and then RPMI-1640 medium was added for future use. SORA and CRIZ powders (SORA:CRIZ = 2:1 ratio) were added to the DMSO solution, then RPMI-1640 medium was added and mixed well for subsequent usage. A 96-well plates were filled with 3 × 10^6^ 4T1 and A549 cells, which were then cultured at 37 °C in a humidified incubator with 5% CO_2_ for 24 h. They were then exposed to PECE (100–1000 µg/mL), free drug (SORA + CRIZ for 1–10 mg/mL) and SORA–CRIZ@NPs (1–10 mg/mL) for 72 h. After 3–4 h, 20 µL of MTT (3 mg/mL) was added to each well and incubated. This was followed by shaking each well for about 10 min. The absorbances of each well were measured at 490 nm using an iMark microplate absorbance reader (Synergy 2, Biotek, Winooski, VT).

### 2.7. Screening of morphological changes

The acridine orange/ethidium bromide (AO/EB) method was modified slightly from that previously reported. In a 48-well plate with RPMI-1640 medium, 1 × 10^3^ of 4T1 and A549 cells were plated in each well. Ten micrograms per milliliter of both AO and EB were incubated for five minutes. An apoptotic assay was performed after treatment with blank PECE, free drug (SORA + CRIZ), and SORA–CRIZ@NPs. Through AO/EB staining, live and dead cells will be stained with AO, whereas dead cells with EB will be spoiled with EB once they have been penetrated (Mohamed Subarkhan et al., 2016; Subarkhan & Ramesh, 2016; Mohamed Kasim et al., 2018; Mohamed Subarkhan et al., 2019). Greenish, yellowish, reddish-orange fluorescence emitting cells based on living, necrosis, and late apoptosis of cells will be recorded using a fluorescent microscope (Leica inverted Fluorescence Microscope SP5 II HCS A, Wetzlar, Germany).

4T1 and A549 cells were maintained in 48-well plates. DAPI (4′,6-diamidino-2-phenylindole) was used to determine the number of dead cells and nuclear morphology after blank PECE, free drug (SORA + CRIZ), and SORA–CRIZ@NPs treatment for 24 h. After the treatment, 10 µg/mL of DAPI was produced in PBS and given to the treated cells. It entered the dead cell membrane and stained the nucleus, but it did not permeate the dead cell membrane. In other words, 10 µg/mL cannot penetrate through viable cell membranes (Mohan et al., 2018; Balaji et al., 2020; Sathiya Kamatchi et al., 2020). The nuclear morphology of the cells will be recorded using a fluorescent microscope (Leica inverted Fluorescence Microscope SP5 II HCS A, Wetzlar, Germany).

### 2.8. Examination of the mode of cell death

A maximum of 4 × 10^3^ cells per well was plated onto six-well plates and incubated for 24 hours. A new medium containing blank PECE, free drug (SORA + CRIZ) and SORA–CRIZ@NPs was then used. The control group consisted of 4T1 and A549 cells that had not been treated in any way. The incubation period was 3 h, followed by three PBS rinses before the cells were removed. After trypsin digestion, the cells were centrifuged at 100×*g* for 10 minutes. A final analysis using flow cytometry was performed on resuspended cells (CytoFLEX; Beckman Coulter, Brea, CA) (Saha et al., 2003; Pandurangan et al., 2016; Sanad et al., 2020).

### 2.9. Assessment of *in vivo* anti-tumor efficacy

An animal model has been developed by injecting 4T1 cell suspension via hypodermic injection. Every other day, the mice's body weights and tumor volumes were measured. Four groups of mice were randomly divided after the tumor volume reached 65 mm^3^: group I: saline, group II: blank PECE, group III: SORA + CRIZ (0.1% of DMSO, SORA: 10 mg/kg, CRIZ: 5 mg/kg of the ratio of SORA/CRIZ = 2/1), and group IV: SORA–CRIZ@NPs group (8.50 mg/mL, SORA: 10 mg/kg, CRIZ: 5 mg/kg of the ratio of SORA/CRIZ = 2/1). Every two days, mice were injected with 200 µL of a drug into their tail veins, including saline. Saline solution used as a control. Four mice in each group were sacrificed after the five treatments, and the tumor tissues were collected.

### 2.10. Assessment of histology

Ki-67 staining and TUNEL reagent were used for immunohistology and apoptosis assays, respectively. Microscopes were used to examine and image the slides. Using 4% formalin, both the cell samples and the tumor tissues were fixed before staining. The samples were then incubated and stained according to the commercial TUNEL FITC Apoptosis Detection Kit (Vazyme Biotech Co., Ltd., Nanjing, China).

### 2.11. Assessment of *in vivo* systemic toxicity

Following completion of *in vivo* antitumor experiments, the animals' organs (heart, spleen, liver, lungs, and kidney) and tumors were removed from the mice, fixed in 4% paraformaldehyde solution for 24 h, embedded in paraffin, cut into 8-µm-thick sections, stained with H&E, and examined under an optical microscope (Leica inverted Fluorescence Microscope SP5 II HCS A, Wetzlar, Germany).

### 2.12. Statistical analysis

We employed the Graph Prism 8.0 software (La Jolla, CA) to accomplish statistical analysis. All data were conveyed as mean ± standard deviation (SD). Student's *t*-test was applied to verify the statistical significance of differences, and *p*< .05 was considered statistically significant.

## 3. Results and discussion

### 3.1. Fabrication and characterizations of SORA–CRIZ@NPs

**Figure 3.**
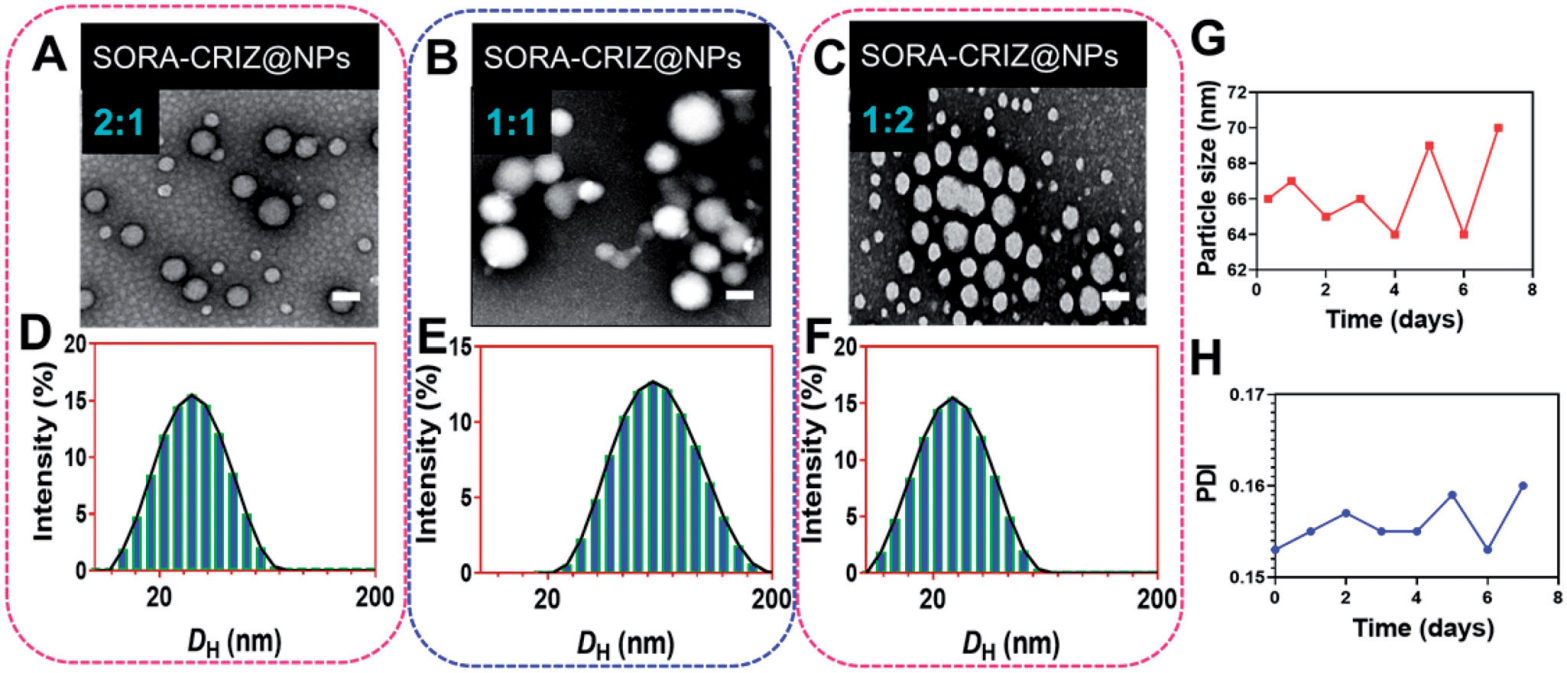
Characterization of nanoparticles. (A–C) TEM image of SORA–CRIZ@NPs with various composition (2:1, 1:1, and 1:2). The scale bar = 100 nm. (C, D) DLS analysis of respective nanoparticle composition. (G, H) Stability of the SORA–CRIZ@NPs for seven days.

The polymers were synthesized via molten ring-open copolymerization in this examination, which yielded a triblock PECE copolymer (Ruttala et al., 2017; Lajous et al., 2018; Sun et al., 2018). The mass ratios of SORA/CRIZ (2:1, 1:1, and 1:2) were used to fabricate the SORA–CRIZ@NPs by self-assembly and confirmed by the particle size, PDI, and zeta-potential of the SORA–CRIZ@NPs. The structure of the NPs with various compositions was confirmed by the TEM analysis (Figure 3(A–C)). Particle size of SORA/CRIZ (2:1, 1:1, and 1:2) was 30.25 ± 1.78, 62.58 ± 2.17, and 27.89 ± 1.92, respectively (Figure 3(D–F)). PDI of SORA/CRIZ (2:1, 1:1, and 1:2) was 0.157 ± 0.005, 0.105 ± 0.012, and 0.210 ± 0.014, respectively. Zeta-potential of SORA/CRIZ (2:1, 1:1, and 1:2) was −18.25 ± 0.21, −7.52 ± 0.52, and −4.91 ± 0.43, respectively. SORA–CRIZ@NPs showed the low particle size distributions of SORA–CRIZ@NPs. SORA–CRIZ@NPs suggested the negative potential surface (−18.25 ± 0.21 mV) of SORA–CRIZ@NPs. Hence, SORA–CRIZ@NPs with a SORA/CRIZ ratio (2:1) was chosen for further *in vivo* and *in vitro* investigations. DLA of SORA and CRIZ was 13.52% and 7.2%, respectively, and the EE was about 93.2% and 91.2%. Further, we assessed the stability of the SORA–CRIZ@NPs for seven days, which shows no remarkable changes during the investigation.

**Figure 4.**
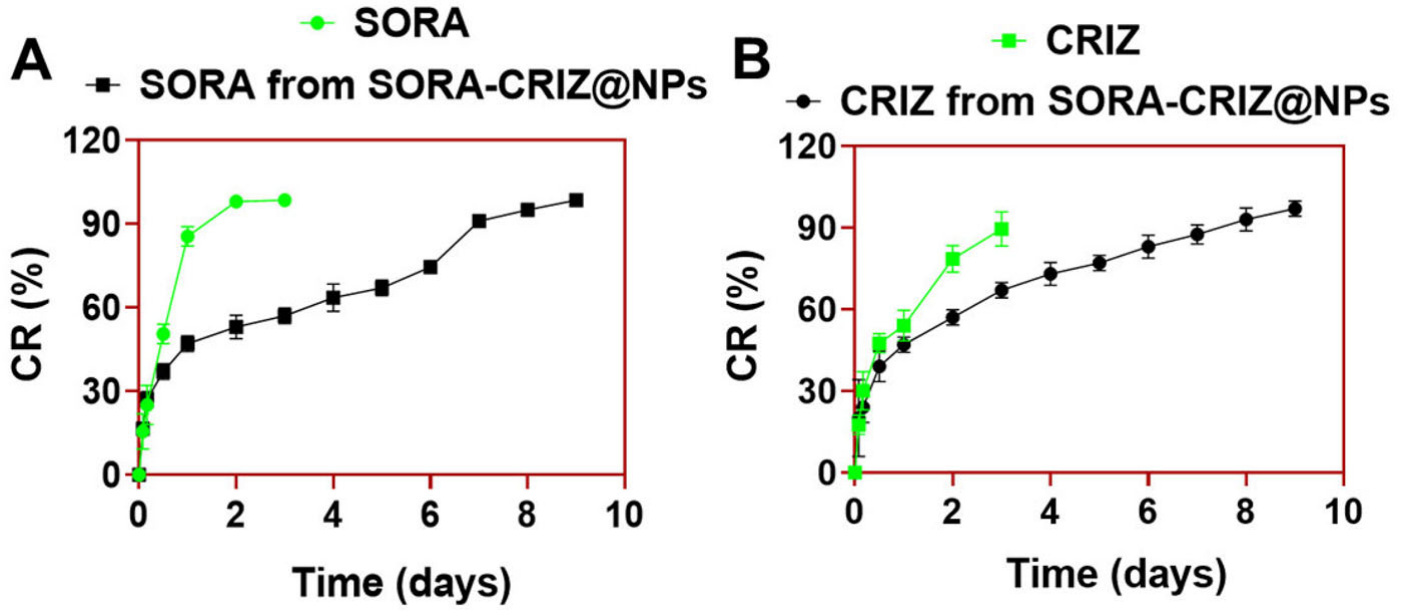
*In vitro* (A) SORA release profiles from SORA–CRIZ@NPs and (B) CRIZ release manners from SORA–CRIZ@NPs in PBS (0.5%) at pH 7.4.

From the SORA and CRIZ, the SORA–CRIZ@NPs release profiles are shown in Figure 4. According to the investigation, more than 82% of free SORA was discharged within two days (Figure 4(A)). About 84% of free SORA was discharged from SORA–CRIZ@NPs for eight days, indicating that SORA was released slowly and steadily from the drug delivery system. More than 90% of free CRIZ was discharged into the medium after three days in contrast to the slow release of free CRIZ. Within eight days, roughly 82% of CRIZ was slowly released from SORA–CRIZ@NPs (Figure 4(B)).

### 3.2. *In vitro* cellular uptake

**Figure 5.**
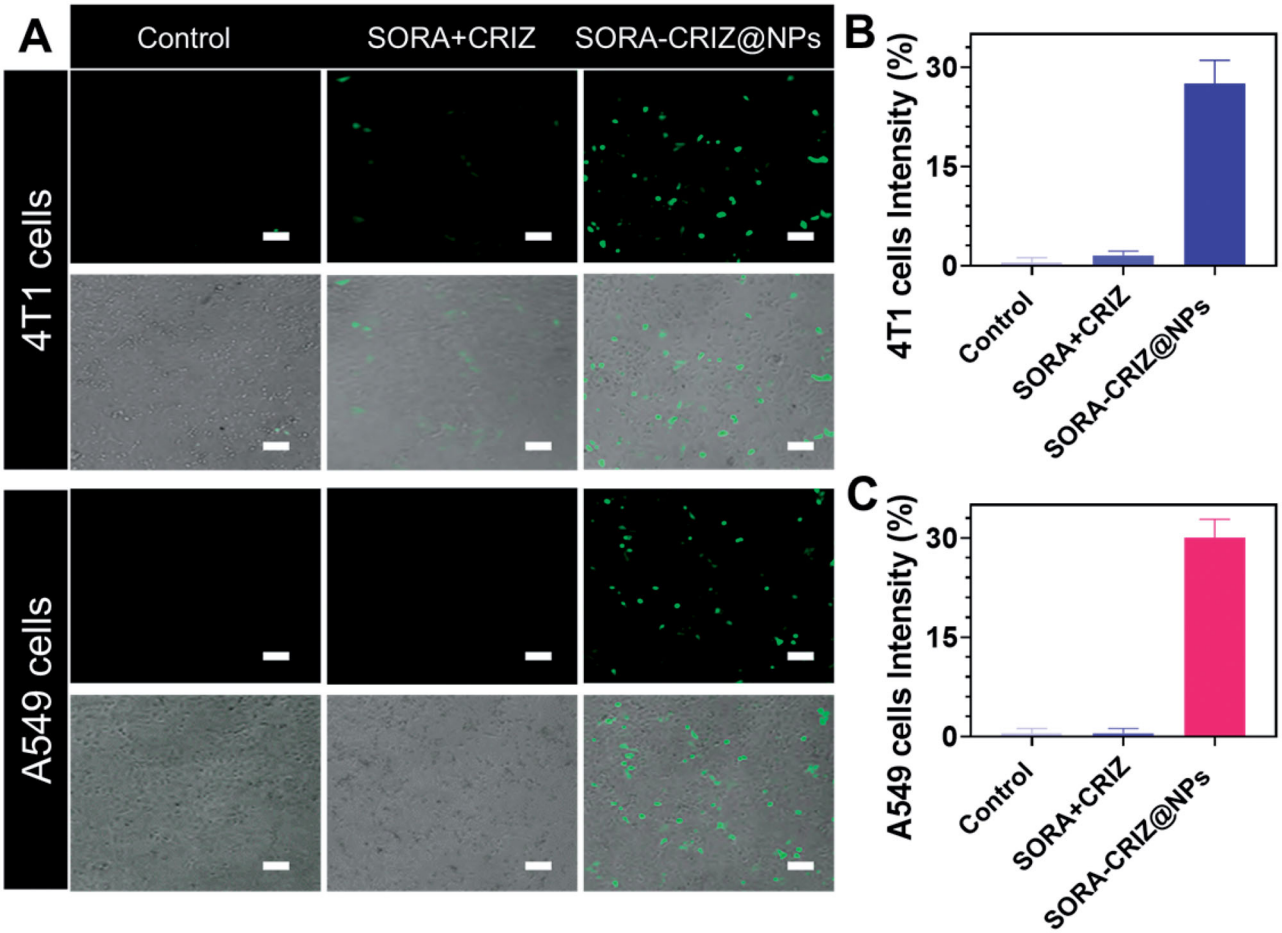
(A) *In vitro* cellular uptake property of SORA + CRIZ and SORA–CRIZ@NPs for 2 h incubation. (B, C) Percentage of the intensity. The scale bar = 50 µm.

SORA–CRIZ@NPs were evaluated *in vitro* for cellular uptake using the fluorescence features, as shown in Figure 5(A). (SORA + CRIZ) powder and SORA–CRIZ@NPs were incubated for 2 h with 4T1 and A549 cells. In the control group, no fluorescence signal was observed (Figure 5(B,C)). A stronger fluorescence signal was observed in cells treated with SORA–CRIZ@NPs (Figure 5) than SORA + CRIZ, suggesting that the hybrid drug payload with PECE-NPs was more quickly absorbed 4T1 and A549 cancer cells.

### 3.3. Assessment of *in vitro* cytotoxicity

**Figure 6.**
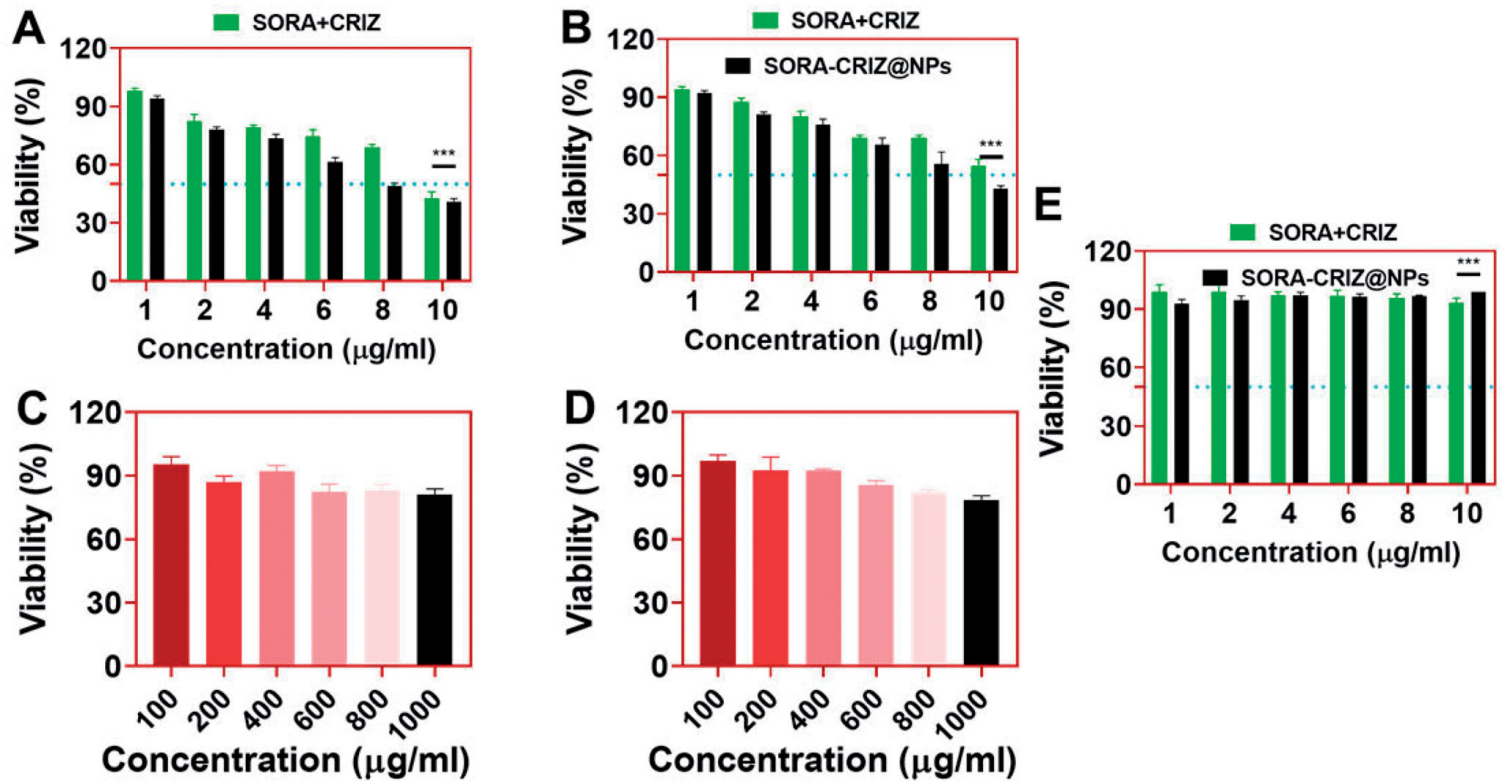
*In vitro* cytotoxicity of (A) A549 and (B) 4T1 cells in the presence of SORA + CRIZ and SORA–CRIZ@NPs with various concentrations for 72 h incubation. *In vitro* viability of (C) A549 and (D) 4T1 cells in the presence of blank PECE-NPs for 72 h incubation. (E) Non-cancerous HUVEC cells in the presence of SORA + CRIZ and SORA–CRIZ@NPs with various concentrations for 24 h incubation (data represent mean ± SD, *n* = 6, Student’s *t*-test, ***p<.005).

The drug delivery system of SORA–CRIZ@NPs was incubated with 4T1 and A549 cells for 72 h. Both treatments suppressed 4T1 and A549 cells in a dose-responsive method associated with the control group. SORA–CRIZ@NPs had greater viability than SORA–CRIZ@NPs at all examined concentrations (Figure 6(A,B)). SORA and CRIZ are released slowly from the nanoparticulate drug delivery system, which might cause the examination.

A549 and 4T1 cells were also investigated for cytotoxicity of the blank PECE-NPs (Figure 6(C,D)). Despite a modest decrease in cell viability with the rise in the concentrations of blank PECE-NPs, more than 82% viable cells at the concentrations of 1000 μg/mL (Figure 6), suggesting that might be predicted to be effective with the SORA–CRIZ@NPs. Therefore, it might be hypothesized that the SORA–CRIZ@NPs could be an efficient drug delivery system. Further, we examined the non-cancerous cells in the presence of SORA + CRIZ and SORA–CRIZ@NPs with various concentrations for 24 h incubation, which shows no significant reduction confirms that the NPs did not affect non-cancerous cells (Figure 6(E)).

### 3.4. Screening of morphological changes

**Figure 7.**
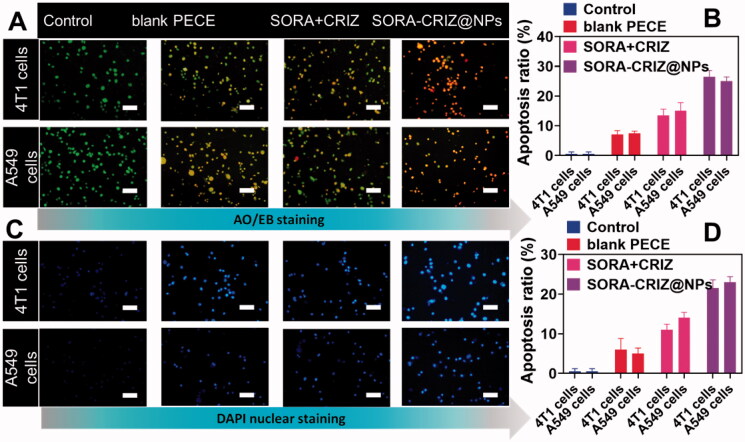
Morphological screening of control, blank PECE, SORA + CRIZ, and SORA–CRIZ@NPs. (A, B) AO/EB staining and respective apoptosis ratio. The scale bar = 100 µm. (C, D) DAPI staining with respective apoptosis ratio. The scale bar = 100 µm.

Acridine orange and ethidium bromide, fluorescent DNA binding dyes, are used in combination with the morphological characteristic of chromatin condensation in stained nuclei. For IC_50_ concentrations of blank PECE, free drugs (SORA + CRIZ) and the SORA–CRIZ@NPs, the AO/EB test may be used to determine their 4T1 A549 cells membrane destabilizing potential. When SORA–CRIZ@NPs were applied to the 4T1 and A549 cells, the number of viable and apoptotic cells increased, as well as the number of necrotic cells (Figure 7(A,B)). Compared with the control (98%), cells treated to free drugs (SORA + CRIZ) and the SORA–CRIZ@NPs exhibited significant decrease in the number of viable cells and an increase in the percentage of early apoptotic cells (Kasibhatla et al., 2006; Liu et al., 2015; Zhang et al., 2019).

To investigate, the nuclear damages of the 4T1 and A549 cells were treated for 24 h with IC_50_ concentrations of blank PECE, free drugs (SORA + CRIZ) SORA–CRIZ@NPs. Cytological alterations were observed using DAPI staining to observe the cytological changes that occurred. Apoptosis-related alterations in SORA–CRIZ@NPs such as chromatin fragmentation and bi- and multi-nucleation are illustrated in Figure 7(C). The number of aberrant cells rises, demonstrating that SORA–CRIZ@NPs cause cytological alterations. The SORA–CRIZ@NP is a very successful drug because it promotes the death of cancer cells by causing the death of cancer cells by inducing the death of 4T1 and A549 cancer cells by inducing the death of cancer cells (Figure 7(C,D)).

### 3.5. Assessment of cell death by flow cytometry analysis

**Figure 8.**
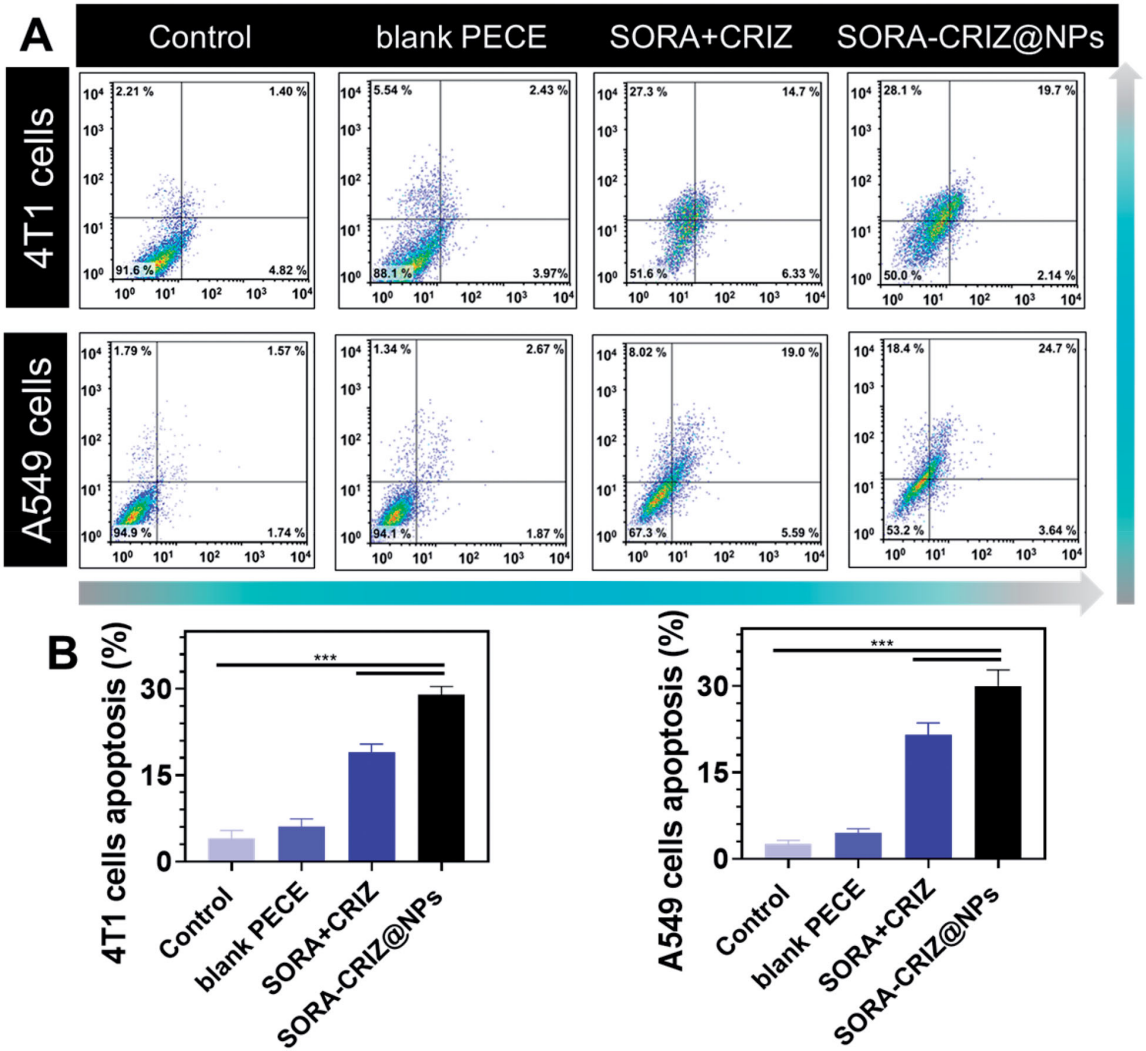
(A) Apoptosis investigation of control, blank PECE, SORA + CRIZ, and SORA–CRIZ@NPs with A549 and 4T1 cells. (B) Apoptosis ratio of 4T1 and A549 cells (data represent mean ± SD, *n* = 6, Student’s *t*-test, ***p<.005).

The mechanism of apoptosis was evaluated by flow cytometry analysis, as previously described (Figure 7). The results obtained by AO/EB and nuclear staining fluorescence microscopy were comparable with flow cytometry (Figure 8). Compared with control, 4T1 and A549 cells treated with blank PECE, free drugs (SORA + CRIZ), SORA–CRIZ@NPs, SORA–CRIZ@NPs exhibited a higher rate of apoptosis, indicating that the NPs successfully induced cell death by 4T1 and A549 cells. The lower apoptosis rate was found in the blank PECE, free drugs (SORA + CRIZ) compared with the NPs. These findings are consistent with those of the *in vitro* anticancer activity assay, supporting the superior cytotoxic activity of SORA–CRIZ@NPs through the apoptotic mode of the cell death pathway (Figure 8(A,B)). Thus, we speculate that SORA–CRIZ@NPs based on PECE-NPs as a targeting molecule have great potential as vehicles for anticancer drug delivery systems.

### 3.6. Assessment of *in vivo* anti-tumor effect

**Figure 9.**
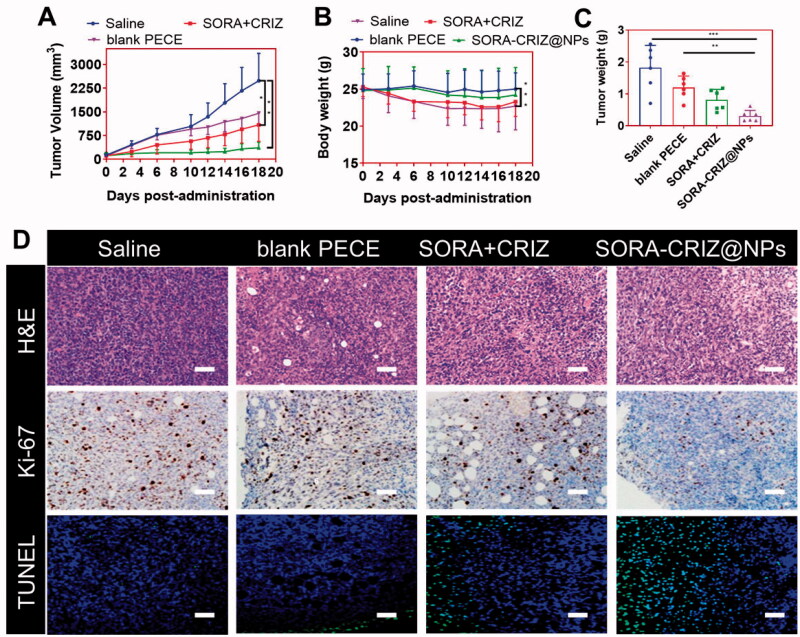
*In vivo* evaluation of the antitumor efficacy of saline, blank PECE, SORA + CRIZ, and SORA–CRIZ@NPs. (A) Tumor growth curves of mice by various treatments. (B) The body weights of mice by various treatments. (C) Average tumor weight for each group. (D) H&E, Ki-67, and TEUNEL staining of respective treatment groups. The scale bar = 100 µm. *p<.05, **p<.01, and ***p<.005 (two-tailed Student’s *t*-test).

As shown in Figure 9(A), the 4T1 tumor volumes of four groups were evaluated frequently and noticed. SORA–CRIZ@NPs caused the slowest growth in tumor volume among the rest of the groups. As the trial progressed, this propensity persisted. A substantial reduction of tumor development was seen in comparison to the rest of the three groups. Like the standard saline group, Figure 9(C) shows that the tumor developments were not affected by the treatments with the group of blank PECE-NPs. In Figure 9(B), the mice's body weights were measured every other day observed a significant difference among each group at the start of the treatment. SORA + CRIZ group mice continued to lose weight, which might be related to the adverse effects of SORA and CRIZ, according to the study's findings.

In contrast, the weight of the remainder of the groupings increased steadily with time. As a result of the delivery of SORA–CRIZ@NPs, the mice's weight loss was effectively stopped. The saline group (18 days) and blank PECE group (18 days) did not significantly differ in median survival time. 4T1 tumor-bearing mice who received SORA–CRIZ@NPs lived longer (18 days) than mice in the free drug group (SORA + CRIZ). This suggests that the drug delivery system might be used in a clinical setting with improved therapeutic efficacy and decreased toxicity.

### 3.7. Assessment of histology

Compared to the other groups, the SORA–CRIZ@NPs group had fewer Ki-67-positive cells in its tumor tissues (Figure 9(D)). There were significantly more Ki-67-positive cells in the saline group than in the blank PECE group. As compared to the other groups, SORA–CRIZ@NPs treatment resulted in a significant reduction in Ki-67 positive cells. Alternatively, no remarkable difference in the Ki-67 positive cell populations was detected among the saline and blank PECE groups (Figure 9(D)). For cell apoptosis, TUNEL examination was used to determine the SORA–CRIZ@NPs might induce cell death in tumor tissues. Compared to other groups, the number of apoptotic (TUNEL positive) cells was more significant in the SORA–CRIZ@NPs group than in the rest groups. Figure 9(D) reveals that the SORA–CRIZ@NPs group had a greater apoptosis index than the other groups. Based on the study results, the SORA–CRIZ@NPs might limit tumor cell growth by stimulating the apoptotic process.

### 3.8. Assessment of *in vivo* toxicity of SORA–CRIZ@NPs

**Figure 10.**
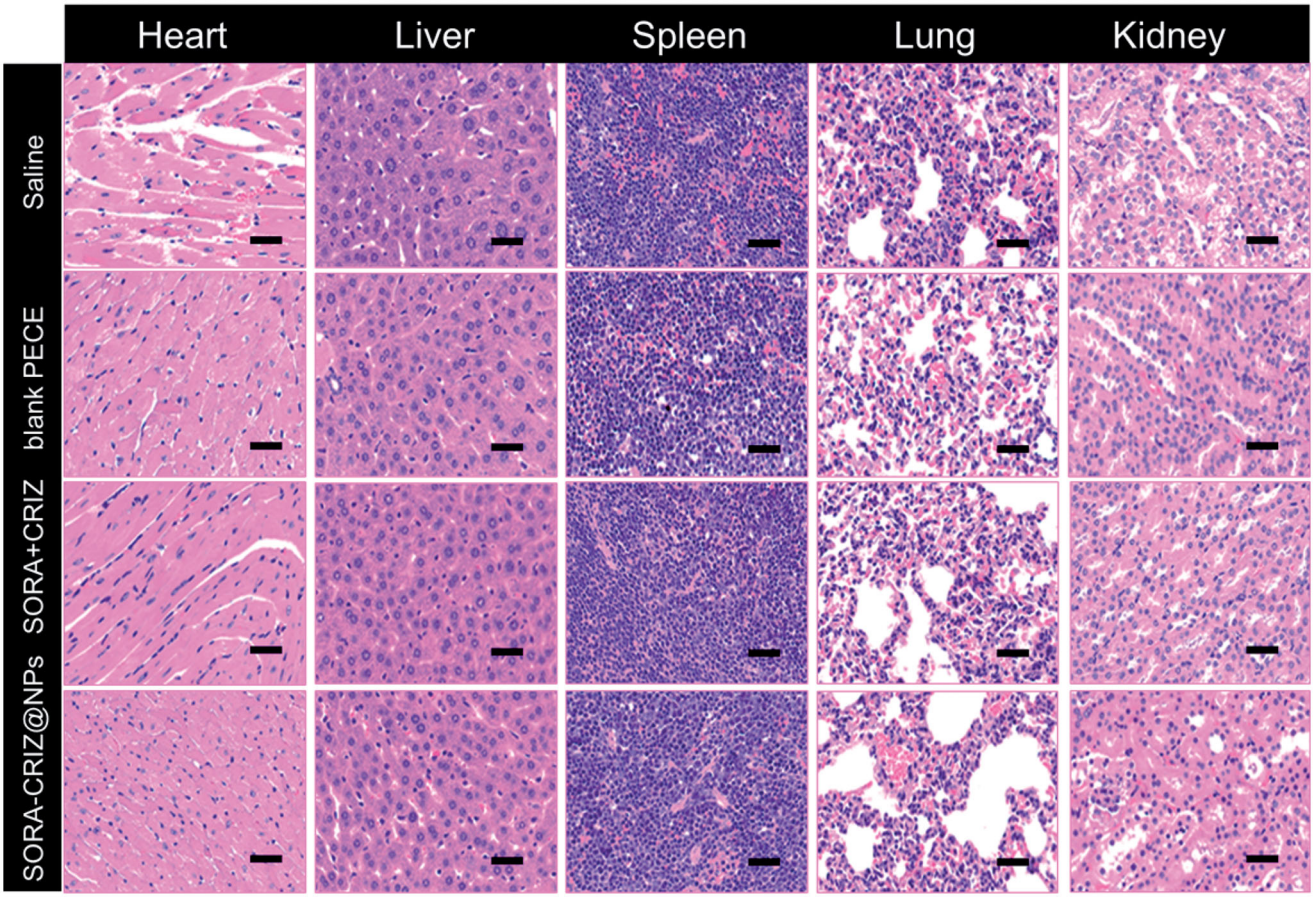
H&E staining of various organs of 4T1 tumor-bearing mice treated with saline, blank PECE, SORA + CRIZ, and SORA–CRIZ@NPs. The scale bar = 50 µm.

SORA–CRIZ@NPs did not produce any significant side-effects in any treatment groups, based on the observation of no aberrant changes in diet or behavior. As part of the study, H&E tissue staining of the slices of several critical organs (heart, liver, lung, kidney, and spleen) of mice was performed. No lesions were observed in the lungs, kidneys, or hearts of any of the treatment groups in Figure 10. However, the treatment of SORA + CRIZ and SORA–CRIZ@NPs to mice resulted in primary pathological alterations in hepatic tissues, including balloon formation. All in all, the data show that SORA–CRIZ@NPs cause very moderate liver damage *in vivo*.

## 4. Conclusions

In conclusion, PECE NPs for the effective co-delivery of SORA and CRIZ have been fabricated in this present investigation. The fabricated NPs displayed small particle size, spherical shape, and narrow particle size distribution, making them acceptable for intravenous administration. The drug delivery system of SORA–CRIZ@NPs released SORA and CRIZ in a sustained way, according to an *in vitro* drug release investigation. Toxicities were dose-dependent, and cell uptake was increased compared to the control experiment when these NPs were administered to 4T1 and A549 cells. The apoptosis pathway was implicated in the cytotoxicity of SORA–CRIZ@NPs on 4T1 and A549 cells. Additionally, the SORA–CRIZ@NPs may effectively suppress tumor development *in vivo* with more extended survival of the animal, lower the glucose metabolisms of tumors and produce minor damage toward essential organs. According to the examination findings, it is possible to treat lung cancer using PECE NPs co-loaded with hybrid drugs (SORA and CRIZ).
